# Axillary Pathological Complete Response After Neoadjuvant Therapy in cN1–2 Breast Cancer: An Internally Validated PET/CT-Integrated Nomogram

**DOI:** 10.3390/curroncol32120667

**Published:** 2025-11-28

**Authors:** Mustafa Berkeşoğlu, Gözde Arslan, Ferah Tuncel, Cumhur Özcan, Zehra Pınar Koç, Pınar Pelin Özcan, Erkan Güler, Sami Benli, Yüksel Balcı, Kadir Eser

**Affiliations:** 1 Department of General Surgery, Faculty of Medicine, Mersin University, 33110 Mersin, Türkiye; 2 Department of Pathology, Kastamonu Training and Research Hospital, 37000 Kastamonu, Türkiye; 3 Department of Pathology, Faculty of Medicine, Mersin University, 33110 Mersin, Türkiye; 4 Department of Nuclear Medicine, Faculty of Medicine, Mersin University, 33110 Mersin, Türkiye; 5 Department of Radiology, Faculty of Medicine, Mersin University, 33110 Mersin, Türkiye; 6 Department of Medical Oncology, Faculty of Medicine, Mersin University, 33110 Mersin, Türkiye

**Keywords:** axillary lymph node, de-escalation, neoadjuvant therapy, nomogram, PET/CT, PERCIST, sentinel lymph node, triple-negative breast cancer

## Abstract

We aimed to develop and internally validate a nomogram to estimate axillary pathological complete response (pCR, ypN0) after neoadjuvant systemic therapy (NAST) in clinically node-positive (cN1–2) breast cancer. In a single-center retrospective cohort of 144 consecutive patients treated with NAST (anti-HER2 as indicated), all underwent standardized pre- and post-NAST 18F-FDG PET/CT and axillary staging (sentinel lymph node biopsy [SLNB], targeted axillary dissection [TAD], or axillary lymph node dissection [ALND]). Axillary pCR occurred in 51.4% (74/144). In a multivariable analysis, independent positive determinants of ypN0 included the triple-negative subtype, Modified PERCIST (SUVmax-based) reduction ≥ 80.70%, pre-NAST tumor-to-axilla SUVmax ratio ≥ 1.21, and residual breast tumor size < 0.5 mm; conversely, conglomerate/matted nodal morphology at diagnosis was inversely associated. The model showed good internal discrimination (AUC 0.857, 95% CI 0.797–0.917) and acceptable calibration (Hosmer–Lemeshow *p* = 0.425). Exploratory, subtype-restricted signals were observed for inflammatory indices within Luminal B (HER2+) but were not retained in the final model. The resulting nomogram—combining tumor biology, PET/CT response, and pre-NAST nodal features—may support risk stratification for axillary de-escalation after NAST; however, prospective external validation—ideally embedded in ongoing de-escalation frameworks—remains essential before clinical implementation, and the tool should currently be regarded as hypothesis-generating rather than a stand-alone decision aid for routine practice.

## Simple Summary

Many women with breast cancer have cancer cells in the armpit (axillary) lymph nodes at the time of diagnosis and receive chemotherapy and targeted drugs before surgery. This treatment can sometimes eliminate the cancer from the nodes, but many patients still undergo extensive lymph node removal, which may cause arm swelling, pain, and reduced shoulder movement. We studied 144 women with breast cancer who were treated before surgery and had scans using positron emission tomography combined with computed tomography both before and after treatment. All patients also had lymph node surgery, so the true status of the armpit could be confirmed via detailed laboratory examination of the removed tissue. We found that tumor type, a strong treatment response on imaging, a favorable balance of uptake between the breast tumor and the armpit nodes before treatment, and very small or no tumor left in the breast after treatment were strongly linked to having no cancer left in the armpit nodes. Using these factors, we created a simple score that estimates the chance of achieving an armpit with no remaining cancer and may help treatment teams avoid unnecessary lymph node surgery in selected patients once it is tested in independent centers.

## 1. Introduction

Breast cancer was the most frequently diagnosed malignancy among women in 2022 (≈2.3 million new cases; 11.6% of all cancers) and remained the leading cause of cancer-related death among women (6.9% of cancer mortality) [1].

Neoadjuvant systemic therapy (NAST) has increasingly been adopted as the initial approach because it reduces tumor and nodal burden and yields prognostic information to guide adjuvant therapy. This strategy has increased axillary pathological complete response (pCR, ypN0) rates—especially in clinically node-positive (cN1–2) disease [2,3,4,5]. Accurate prediction of axillary pCR after NAST is critical, since achieving ypN0 can support omission of axillary lymph node dissection (ALND) and thereby reduce lymphedema, shoulder dysfunction, and sensory morbidity [4,5,6,7,8,9,10]. Despite modern techniques (sentinel lymph node biopsy, SLNB; targeted axillary dissection, TAD), overtreatment persists: approximately 15% of patients who attain axillary pCR still undergo ALND [11].

Current guidelines recommend imaging- and biopsy-based assessment of axillary lymph nodes (ALNs) at diagnosis and after NAST [12,13]. However, axillary ultrasound (US) alone shows limited accuracy for residual disease after NAST, as suggested by preliminary data from the prospective, multicenter AXSANA registry [14]. Accordingly, axillary evaluation is shifting from one-size-fits-all algorithms to individualized, response-guided decision making [15]. Predictive nomograms and risk scores can facilitate this shift, yet tumor heterogeneity, subtype-specific response, and imaging constraints remain substantial challenges [3,16].

Integrating functional imaging—especially 18F-fluorodeoxyglucose positron emission tomography/computed tomography (18F-FDG PET/CT, hereafter PET/CT)—with additional biomarkers has shown promise for improving axillary response prediction [17,18]. Recently, routine inflammatory indices (e.g., neutrophil-to-lymphocyte ratio, NLR; monocyte-to-lymphocyte ratio, MLR; platelet-to-lymphocyte ratio, PLR) have also been explored, though findings are inconsistent [18,19,20].

To investigate these gaps, we retrospectively evaluated clinical, pathological, PET/CT-based, and hematological predictors of axillary pCR in cN1–2 breast cancer treated with NAST and constructed and internally assessed a nomogram to enable individualized axillary management while minimizing unnecessary ALND.

## 2. Materials and Methods

### 2.1. Study Cohort and Eligibility Criteria

This retrospective cohort included female patients aged ≥ 18 years diagnosed with invasive breast cancer at Mersin University Hospital between January 2018 and March 2025. Eligibility required cT1–T4a-c, cN1–N2, M0 disease per the 8th American Joint Committee on Cancer (AJCC) edition. Pre- and post-NAST PET/CT were required. Patients received anthracycline–taxane–based NAST; anti-HER2 therapy was given as indicated. Exclusion criteria included previous ipsilateral axillary surgery, inflammatory breast cancer (T4d), stage IV disease, bilateral breast cancer, and histologic types other than invasive ductal or lobular carcinoma. The primary endpoint was axillary pCR (ypN0). Patients were classified as ypN0 (axillary pCR, including pN0(i+) with isolated tumor cells only or non-ypN0 (any residual macrometastasis, micrometastasis, or extracapsular extension). The study was approved by the institutional ethics committee (2025/459); written consent was waived due to its retrospective nature and anonymized data handling.

### 2.2. Clinical Staging and Imaging

Baseline demographic, clinical, imaging, and pathological parameters were retrospectively recorded, including age, body mass index (BMI), tumor size/location, clinical T-stage, and axillary node status (mobile vs. conglomerate). Tumor size was measured by baseline (pre-NAST) ultrasound and by pathology post-NAST (ypT). Estimated tumor size reduction (%) was calculated from pre- to post-NAST measurements [21].

Clinical nodal status at diagnosis was determined via physical examination and imaging according to AJCC (cN1: mobile nodes; cN2: fixed/matted). Independently, pre-NAST axillary morphology during ultrasound was categorized into three groups: <4 non-conglomerate abnormal nodes, ≥4 non-conglomerate abnormal nodes, and conglomerate/matted nodes. Nodes were deemed radiologically suspicious if they met pre-specified sonographic criteria (e.g., cortical thickness > 3 mm, short-axis ≥ 10 mm, loss of hilum, eccentric cortical thickening, round shape, or abnormal vascularity), in which case US-guided biopsy was performed when feasible.

Patients fasted ≥ 6 h (capillary glucose < 200 mg/dL) before 18F-FDG injection. Whole-body acquisition was performed ≈60 min post-injection from the skull base to mid-thigh using a Siemens Biograph mCT 20 system (Siemens Healthineers, Erlangen, Germany) in 3D mode, with 2–3 min per bed position. A low-dose, non-contrast CT scan was obtained for attenuation correction, scatter correction, and anatomical co-registration. PET images were reconstructed with an iterative ordered-subset expectation maximization algorithm according to the manufacturer’s standard oncologic protocol, and SUVmax values (normalized to body weight) were measured on a dedicated workstation. For axillary measurements, we recorded the SUVmax of the index axillary node (i.e., the lymph node with the highest FDG uptake); “axilla SUVmax” refers to this index-node value. Modified PERCIST (SUVmax-based) reduction (%)—the percent decrease in primary-tumor SUVmax from pre- to post-NAST—was calculated on paired scans [22,23]. We evaluated a novel metric, the Modified Total Metabolic Burden Ratio (Modified PET_MB Ratio), defined as the ratio of the post-NAST sum of tumor SUVmax and axilla SUVmax to the corresponding pre-NAST sum of tumor SUVmax and axilla SUVmax; this composite captures the two-compartment metabolic response (breast tumor and axilla).

### 2.3. Pathology and Immunohistochemistry

Tumors were histologically classified as invasive carcinoma of no special type (NST), “pure” special-type carcinomas (≥90% of a single histologic pattern), or mixed-type carcinomas. Tumors with favorable prognostic morphology (e.g., mucinous, cribriform, papillary, tubular) were analyzed separately. Tumor focality was categorized as multifocal, multicentric, or satellite lesions.

Surgical specimens (breast and axilla) were processed per routine protocols (formalin-fixed, paraffin-embedded; hematoxylin and eosin (H&E); immunohistochemistry (IHC) as needed). ER and PR followed ASCO/CAP (positive if ≥1%); HER2 was scored per ASCO/CAP (IHC 3+ or IHC 2+/ISH+ as positive; IHC 0/1+ or IHC 2+/ISH− as negative; HER2-low noted descriptively) [24,25]. Ki-67 was recorded and summarized by conventional cutoffs (≤5%, 6–29%, ≥30%) to support surrogate subtyping (Luminal A, Luminal B [HER2−/HER2+], HER2-enriched, TNBC) according to published recommendations [25,26]. HRSum (%) was defined as the sum of ER% and PR% measured on IHC (HRSum = ER% + PR%).

### 2.4. Axillary Work-Up and Sentinel Procedure

Axillary imaging included US for all and mammography (MMG); magnetic resonance imaging (MRI) was used selectively. Suspicious nodes underwent US-guided biopsy. From late 2020, proven metastatic nodes were clipped to facilitate targeted excision [27,28].

Axillary management followed contemporary guideline-concordant algorithms: after NAST, patients underwent sentinel lymph node biopsy (SLNB) with dual-tracer mapping (technetium-99m radiocolloid and blue dye) or targeted axillary dissection (TAD) when technically feasible, and completion ALND was recommended when metastatic disease was identified on SLN/TAD pathology (macrometastasis, micrometastasis, or extracapsular extension). For clipped nodes, radioactive occult lesion localization (ROLL) with Tc-99m macroaggregated albumin was used when available. TAD aimed to retrieve ≥3 nodes encompassing clipped and sentinel nodes; excision was guided by gamma probe and dye mapping [28]. Axillary pCR (ypN0) was defined exclusively on surgical pathology after SLNB, TAD, or ALND; PET/CT findings were not used to omit axillary surgery or to define ypN0 in the absence of histologic confirmation.

### 2.5. Intraoperative Assessment and Pathology Definitions

SLNs underwent intraoperative frozen section (H&E). Positive or suspicious findings usually prompted immediate ALND. Final pathology included permanent H&E and ancillary IHC as needed. Nodes with isolated tumor cells (ITCs, <0.2 mm) were coded pN0(i+) and counted as ypN0 for the axillary pCR endpoint, in line with contemporary axillary de-escalation trials in which ITCs are not regarded as clinically relevant residual axillary disease and are treated as node-negative in axillary management algorithms.

### 2.6. Breast Surgery and Response Definitions

Definitive breast surgery consisted of breast-conserving surgery or mastectomy based on tumor response, disease extent, and patient preference; negative margins were required. Residual tumor size (ypT) was measured on final breast pathology and expressed in millimeters (mm), independent of PET/CT findings. Breast pCR was defined as no residual invasive carcinoma (ductal carcinoma in situ [DCIS] allowed), and minimal residual disease categories (including ypT < 0.5 mm) were assigned solely on histopathologic assessment rather than imaging criteria.

### 2.7. Laboratory Assessments and Derived Indices

Peripheral blood samples collected pre-NAST and within one month before surgery were analyzed. Biomarkers included CA 15-3, immature granulocytes (IG), and the post/pre-NAST albumin ratio. Derived inflammatory indices included NLR, MLR, PLR, SII (platelet × neutrophil/lymphocyte), PNI = (10 × serum albumin [g/dL]) + (5 × absolute lymphocyte count), PIV (neutrophil × platelet × monocyte/lymphocyte), HALP = hemoglobin [g/dL] × albumin [g/dL] × lymphocyte count, HRR = hemoglobin/RDW (%), and MPVPLT = MPV (fL)/platelet count.

### 2.8. Outcomes and Statistical Analysis

The primary endpoint was axillary pCR after NAST. Secondary analyses included clinicopathologic/imaging correlates and threshold identification. Continuous variables were summarized as median (IQR) or mean ± SD (per Shapiro–Wilk) and compared using the Mann–Whitney U or *t*-test; categorical variables were determined using the Chi-square or Fisher’s exact test. Receiver operating characteristic (ROC) curves were used to evaluate discrimination and derive optimal cut-offs using the Youden index. Continuous predictors were analyzed as continuous; where a clinically interpretable threshold was required, we derived data-driven cut-offs via ROC/Youden within the study cohort and examined their 2 × 2 association with ypN0. These thresholds were considered exploratory/internal and used for model building and nomogram scaling; no external validation was performed. For subgroup explorations (e.g., Luminal B [HER2+]), ROC directionality reflected biology (TESTPOS = SMALL for NLR/SII/PIV; TESTPOS = LARGE for HALP).

Variables with clinical plausibility and univariate support were entered into multivariable logistic regression after screening for collinearity and missingness. Model calibration and performance were assessed using the Hosmer–Lemeshow test, Nagelkerke R^2^, overall accuracy, and AUC of predicted probabilities. In addition to global AUC, we evaluated operating characteristics at clinically relevant thresholds of the predicted probability of ypN0. Specifically, we calculated sensitivity and specificity for ypN0, the proportion of true ypN0 among patients above a given probability threshold, and the false-negative rate (FNR) for residual axillary disease, defined as the proportion of non-ypN0 patients whose predicted probability of ypN0 exceeded the de-escalation threshold. As an exploratory step, we also examined discrimination of the final multivariable model within the three major surrogate subtype groups (HR+/HER2−, HER2-positive, and TNBC) by assessing ROC curves and AUCs for the predicted probability of ypN0 in each stratum. Given the moderate cohort size and limited per-subtype numbers, we pre-specified a parsimonious multivariable model with a restricted set of predictors and checked events-per-variable to reduce overfitting. The model was designed as a pragmatic prediction tool rather than a fully explanatory subtype-specific framework. Results are reported as adjusted odds ratios (ORs) with 95% confidence intervals (CIs) and two-sided *p* values (*p* < 0.05 significant). Analyses were performed in IBM SPSS Statistics v25 (IBM Corp., Armonk, NY, USA).

## 3. Results

### 3.1. Cohort and Baseline Profile

A total of 144 patients (median age, 51 years; IQR, 43–60) were analyzed. Most tumors were cT2 (58.3%), grade 3 (51.4%), and invasive carcinoma of NST (85.4%). ER-high (≥10%) and PR-high (≥20%) rates were 75.0% and 55.6%, respectively; Ki-67 ≥ 30% in 50.7%; TNBC and HER2-positive subtypes comprised 11.8% and 31.2% (Table 1).

### 3.2. Axillary Treatment and Pathology

Axillary pCR was achieved in 51.4% (*n* = 74). Among non-ypN0 cases, macrometastasis, micrometastasis, and extracapsular extension (ECE) were observed in 52.9%, 7.0%, and 40.0%, respectively. SLNB was attempted in 132 patients (median 3 nodes; IQR 2–3). Management pathways included the following: SLNB alone 40.3% (*n* = 58), completion ALND after SLNB 51.3% (*n* = 74) (predominantly for positive/suspicious frozen-section findings), and direct ALND 8.3% (*n* = 12) (mainly non-identification/mapping failure, technical impediments, or strong clinical–radiologic suspicion). In the ALND-after-SLNB subgroup (*n* = 74), the median total metastatic axillary nodes (SLN + non-SLN) was 2 (IQR 1–7), with ≥3 nodes in 41.9%; the largest metastatic focus was 12 mm (IQR 10–16 mm). Patterns included exclusive SLN (33.8%), isolated non-SLN (“skip”) (8.1%), combined SLN/non-SLN (41.9%), and no metastasis (14.9%); exclusive ECE limited to SLNs was observed in 6.8%. Across all ALNDs (*n* = 86), ypN0 was found in 18.6%, including 33.3% (4/12) within direct-ALND (Table 2).

### 3.3. Baseline Clinicopathologic Correlates of ypN0

Clinical nodal morphology stratified outcomes: the adverse signal was confined to conglomerate/matted nodes (*p* = 0.010), whereas among non-conglomerate disease, having ≥4 versus <4 abnormal nodes did not materially change ypN0 rates. Subtypes favored ypN0 (HER2-positive and TNBC enriched; all *p* ≤ 0.007), and hormone receptor highs were depleted (ER-high/PR-high, both *p* ≤ 0.002). CA 15-3 was modestly lower in ypN0 (*p* = 0.024). Other baseline variables (age, BMI, clinical T stage, grade, laterality, location, Ki-67 category) were balanced (Table 1 and Table 2).

In the overall cohort, routine hematologic indices (NLR, SII, PIV, and HALP) showed no association with axillary pCR (Table 2). In an exploratory analysis limited to Luminal B (HER2+), ypN0 cases had lower NLR/SII/PIV and higher HALP (all *p* ≤ 0.048); ROC discrimination was modest (AUC 0.73–0.78) in terms of Youden thresholds (NLR ≤ 2.35, SII ≤ 609.07, PIV ≤ 373.90, HALP ≥ 39.04). These subtype-restricted signals were hypothesis-generating and were not included in the multivariable model.

### 3.4. Therapeutic and Imaging Correlates

Axillary clearance tracked with systemic response in the breast: ypN0 showed greater pathological tumor size reduction (median 100.0% vs. 40.8%, *p* < 0.001) and higher complete metabolic response on PET/CT (55.4% vs. 24.3%, *p* < 0.001). Imaging surrogates were concordant: pre-NAST, tumor SUVmax was higher, axilla SUVmax was lower, and the tumor-to-axilla SUVmax ratio was higher in ypN0; post-NAST, both tumor and axilla SUVmax were lower in ypN0 (all *p* ≤ 0.004, except pre-NAST axilla *p* = 0.014) (Table 2).

### 3.5. ROC-Derived Thresholds and Categorical Validation

Youden-optimized thresholds yielded pragmatic rules that significantly enriched for ypN0: estimated tumor size reduction ≥ 40.83%, Modified PERCIST reduction ≥ 80.70%, pre-NAST LN size < 19.35 mm, pre-NAST tumor SUVmax ≥ 10.17, pre-NAST tumor-to-axilla SUVmax ratio ≥ 1.21, and post-NAST tumor SUVmax < 2.65 and axilla SUVmax < 1.85 (all *p* ≤ 0.017 in 2 × 2 validation). The post-NAST tumor-to-axilla SUVmax ratio showed no discrimination (*p* = 0.968) and was not pursued further. CA 15-3 was significant for ROC (AUC 0.609; *p* = 0.024) but not in 2 × 2 validation (*p* = 0.064). Overall ROC performance was moderate for key indices (AUC 0.771 for estimated tumor size reduction, 0.694 for Modified PERCIST, and 0.647 for Modified PET_MB Ratio) (Table 3 and Table 4).

### 3.6. Multivariable Model and Nomogram

**Figure 1:**
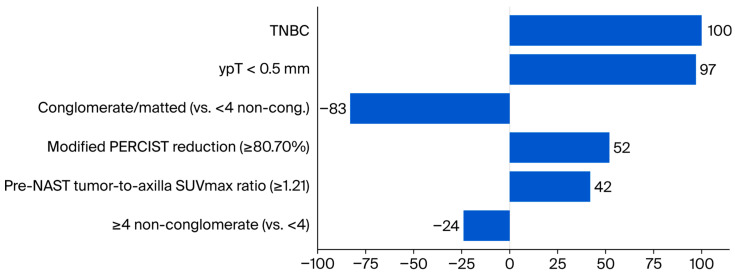
Points-bar contribution plot of predictors of axillary ypN0 derived from the multivariable logistic model; the nomogram and point translation are provided in Table 6. This nomogram displays the relative contribution (points) of the final multivariable predictors to the probability of achieving axillary pathological complete response (ypN0) after neoadjuvant systemic therapy. Positive bars indicate an increased likelihood of ypN0, whereas negative bars indicate a decreased likelihood. The point scale was derived from the logistic model by rescaling coefficients to a 0–100 range (largest absolute coefficient = 100 points). Abbreviations: NAST, neoadjuvant systemic therapy; yp, post-NAST; TNBC, triple-negative breast cancer; SUVmax, maximum standardized uptake value; Modified PERCIST (SUVmax-based) reduction (%), percent reduction in primary tumor SUVmax between pre-NAST and post-NAST PET/CT; non-cong., non-conglomerate.

Independent predictors of ypN0 were TNBC (OR 11.41; 95% CI 2.43–53.54), ypT < 0.5 mm (OR 10.63; 3.51–32.17), Modified PERCIST reduction ≥ 80.70% (OR 3.56; 1.46–8.69), and pre-NAST tumor-to-axilla SUVmax ratio ≥ 1.21 (OR 2.76; 1.13–6.74). These PET/CT-derived predictors were evaluated against a pathology-based reference standard of axillary ypN0 after SLNB, TAD, or ALND, and are therefore intended as preoperative risk markers rather than imaging-only surrogates of nodal clearance. Notably, the predictor “ypT < 0.5 mm” reflects pathology-confirmed minimal residual breast disease rather than PET/CT clinical complete response alone, underscoring that imaging may appear negative while submillimetric invasive foci persist and that our model treats such minimal ypT as a histologic, not purely imaging-derived, correlate of favorable axillary response. Conglomerate/matted morphology was inversely associated (vs. <4 non-conglomerate: OR 0.13; 0.04–0.51), whereas ≥4 vs. <4 non-conglomerate was not significant (OR 0.56; 0.22–1.41). Discrimination and calibration were good (AUC 0.857, 95% CI 0.797–0.917; SE 0.031; *p* < 0.001; Hosmer–Lemeshow *p* = 0.425), with 76.4% accuracy and Nagelkerke R^2^ 0.483 (Table 5). A points-based nomogram derived from these coefficients is shown in Figure 1, with point-to-risk mapping shown in Table 6. The wide confidence interval around the TNBC estimate reflects the small TNBC subgroup and indicates limited precision rather than a definitive, large causal effect. Conversely, HER2-positive and luminal subtypes showed higher ypN0 proportions in univariable analyses but did not retain independent significance once metabolic response, pre-NAST tumor-to-axilla SUVmax ratio, and residual breast tumor size were included, suggesting that much of their favorable behavior is mediated through these response-related variables.

To relate discrimination to de-escalation decisions, we examined the nomogram at two high-probability thresholds corresponding to the >70% and >85% predicted ypN0 risk categories in Table 6 (predicted ypN0 ≥ 0.70 and ≥0.85, respectively). At the ≈70% threshold, n = 49/144 (34.0%) patients were assigned to the high-probability de-escalation group; 44 of these had ypN0 (89.8%), yielding a sensitivity for ypN0 of 59.5%, a specificity of 92.9%, and an FNR for residual axillary disease of 7.1%. At the more stringent ≈85% threshold, n = 34/144 (23.6%) were allocated to the higher-probability de-escalation group; 30 of these had ypN0 (88.2%), with a sensitivity of 40.5%, specificity of 94.3%, and an FNR of 5.7%. These values illustrate the trade-off between reducing the risk of undertreating residual nodal disease and decreasing the proportion of patients eligible for omitting ALND.

In exploratory subtype-restricted analyses, the nomogram maintained good discrimination in HR+/HER2− and HER2-positive disease, whereas estimates were less precise in TNBC. Among HR+/HER2− tumors (Luminal A and Luminal B HER2−; n = 82), the AUC for predicted ypN0 was 0.82 (95% CI 0.73–0.91). In HER2-positive disease (Luminal B HER2+ and non-Luminal HER2+; n = 45), the AUC was 0.90 (95% CI 0.80–0.99). In the smaller TNBC subgroup (n = 17), the AUC was 0.71, with a wide confidence interval (95% CI 0.45–0.98). Given these limited per-subtype sample sizes, particularly for TNBC, we interpret these stratified results as hypothesis-generating and did not construct separate subtype-specific nomograms.

## 4. Discussion

In a cN1–2 cohort treated with NAST, we observed substantial heterogeneity in axillary response. In the multivariable analysis, biology (TNBC), metabolic response (Modified PERCIST reduction ≥ 80.70%), pre-NAST PET/CT tumor-to-axilla SUVmax ratio (≥1.21), and minimal residual breast disease (ypT < 0.5 mm) independently increased the odds of axillary pCR, whereas conglomerate nodal morphology was inversely associated. Across non-conglomerate presentations, having ≥4 versus <4 abnormal nodes was not independently discriminatory, underscoring that morphological conglomeration rather than nodal count alone carries the dominant adverse signal. The model discriminated well (AUC 0.857) and showed acceptable calibration, supporting response-guided, individualized axillary decision making.

Concordance between breast and axillary response has both practical and biological implications. Approximately one-sixth of patients (15.6%) achieving breast pCR still had residual nodal disease, as noted by Flores et al., underscoring the necessity for thorough axillary assessment even in patients achieving breast remission [29].

Higher axillary pCR in TNBC/HER2-positive subtypes and the coupling between breast pCR/minimal ypT and nodal clearance are aligned with prior reports on subtype-specific chemosensitivity and breast–node response concordance [3,16,30]. Our data further support the clinical utility of PET/CT-based response assessment after NAST: greater primary-tumor SUVmax reductions per Modified PERCIST and a higher pre-NAST tumor-to-axilla SUVmax ratio were associated with ypN0, consistent with prior PET/CT studies in which metabolic response correlated with nodal clearance (metabolic complete response in axillary nodes) and informed treatment planning [17,23,31,32,33].

In the Luminal B (HER2+) subset, lower NLR/SII/PIV and higher HALP were associated with ypN0 and yielded modest discrimination in ROC analysis—hypothesis-generating and not incorporated into the final model. This mirrors heterogeneous literature in which inflammatory markers may carry subtype-dependent value and likely perform best when integrated with imaging/clinicopathologic variables rather than used alone [20,34,35].

Although TAD and dual-tracer mapping reduce SLNB false-negative rates [3,7,27], the clinical value of pursuing ever-lower FNRs is debated, given very low axillary recurrence in contemporary series (<2%) [36,37,38]. In this context, we present the nomogram not only by its global AUC but also in terms of its FNR for residual nodal disease, the proportion of true ypN0 among high-probability candidates, and related operating characteristics at clinically plausible thresholds, enabling clinicians to judge whether the residual risk of missed axillary metastases is acceptable in their own practice. Accordingly, neither PET/CT nor our nomogram is proposed as a stand-alone substitute for pathological axillary staging; rather, they are meant to complement standard SLNB/TAD pathways by identifying patients with a higher probability of ypN0 in whom omission of ALND or trial-based de-escalation strategies may be considered, while maintaining SLNB- or TAD-based confirmation of nodal status. Our data still show that technical and intraoperative uncertainties can lead to ALND in a minority of patients; continual improvements in localization and pre-/intraoperative prediction tools should help reserve ALND for those with the highest probability of residual disease.

More than half of the cohort achieved ypN0, and many of the remainder had limited nodal burden, supporting risk-adapted de-escalation strategies. Together with contemporary trials/registries, our findings reinforce the safety signal of selective SLNB-alone in carefully staged/responding patients and the potential role of axillary radiotherapy (RT) as an alternative to completion ALND in selected residual disease scenarios [4,15,36,37,38,39,40,41,42]. Within this de-escalation framework, current guidelines still mandate completion of ALND when residual nodal metastasis is detected on SLNB or TAD after NAST, while emerging evidence supports axillary RT as a potential alternative in carefully staged responders. Even when PET/CT suggests axillary clinical response, SLNB or TAD remains indispensable to assess residual nodal disease in cN0 patients after NAST; within this constraint, response-guided tools that combine subtype, PET/CT metrics, and minimal breast residual may help avoid unnecessary ALND without compromising regional control. Even if ongoing trials establish RT as a preferred alternative to completion ALND in HER2-positive and TNBC patients with breast and axillary ypCR, accurate estimation of axillary ypCR will remain essential for selecting candidates, and our nomogram—developed in a surgically staged cohort—should be regarded as a hypothesis-generating risk stratification tool that will require prospective reassessment in RT-based algorithms.

Our findings support a biologically plausible link between minimal residual breast disease (pathology-confirmed ypT < 0.5 mm) and favorable axillary response after NAST. This aligns with an emerging de-escalation paradigm exemplified by SOUND, where omission of axillary surgery was non-inferior in carefully selected cN0 upfront-surgery patients [5,43]. Direct translation to the post-NAST, initially node-positive setting remains premature given simultaneous breast–axilla interventions and distinct biology [44]. Nonetheless, response-guided strategies—for example, two-staged surgical workflows, standardized PET/CT response readouts, and validated nomograms—warrant prospective testing to identify patients with ypT < 0.5 mm (or comparable very low residual burden) who might safely avoid further axillary surgery. Together with de-escalation trials, a simple ruleset—pre-NAST tumor-to-axilla SUVmax ratio ≥ 1.21, deep metabolic response (≥80.70%), and minimal breast residual—may help prioritize SLNB-alone and avoid unnecessary ALND in carefully staged responders, pending external validation.

Strengths include systematic pre- and post-NAST PET/CT acquisition, consistent pathology review, and a parsimonious multivariable model translated into a practical nomogram. Limitations should temper interpretation: this study is retrospective and single-center with a moderate sample size, lacks external validation and long-term outcomes, and did not incorporate genomic profiling or regimen-level treatment heterogeneity. Accordingly, this PET/CT-integrated nomogram should be viewed as an internally validated, hypothesis-generating model; it is not yet suitable for routine, stand-alone use outside institutions with similar case-mix and workflows until independent external calibration and validation are available. Baseline radiology used mixed modalities (US/MMG ± MRI), so radiologic response was not a primary endpoint; pathology served as the reference. Because per-subtype numbers were modest—especially for TNBC—subtype-restricted performance estimates of the nomogram were imprecise, and we did not attempt subtype-specific recalibration. The current model should therefore not be interpreted as a fully subtype-tailored tool; larger, multicenter cohorts with sufficient subtype-specific events will be required to determine whether dedicated subtype-specific models or recalibration are warranted. Exploratory hematologic markers were subset-restricted and hypothesis-generating. Prospective, multicenter validation—ideally embedding early on-treatment assessments and dynamic biomarker/imaging changes—is needed before routine clinical adoption.

## 5. Conclusions

In cN1–2 breast cancer treated with NAST, axillary pCR was independently associated with tumor biology (TNBC), deep metabolic response on PET/CT (Modified PERCIST reduction ≥ 80.70%), a higher pre-NAST tumor-to-axilla SUVmax ratio (≥1.21), and minimal residual breast disease (ypT < 0.5 mm), whereas conglomerate nodal morphology was inversely associated. Across non-conglomerate disease, nodal count alone was not discriminatory, whereas conglomerate/matted morphology consistently predicted lower odds of ypN0 and therefore carries weight in the nomogram. The resulting model showed good discrimination (AUC 0.857) and acceptable calibration and was translated into a concise nomogram that combines subtype, imaging response, and baseline nodal features to support individualized axillary de-escalation after NAST. Prospective external validation—ideally embedded in ongoing de-escalation frameworks [45,46] (e.g., ALLIANCE A011202, EUBREAST-01)—is essential before routine adoption in other centers, including in settings where axillary RT rather than ALND is increasingly being evaluated for responders. Until such validation is available, this internally validated nomogram should be used as a research and decision-support tool in multidisciplinary discussions, rather than as a clinically confirmed risk calculator for routine use outside our institution. If future trials confirm its performance in RT-integrated de-escalation pathways, the nomogram may contribute to multidisciplinary decisions about omitting ALND in carefully selected patients, while still requiring guideline-concordant axillary staging and long-term outcome data.

## Figures and Tables

**Table 1:** Comparison of categorical clinicopathological and imaging characteristics according to axillary response status (non-ypN0 vs. ypN0).

Variables	Non-ypN0 (*n* = 70)	ypN0 (*n* = 74)	*p*-Value
Age category			0.732
≤50 years	33 (47.1%)	37 (50.0%)	
>50 years	37 (52.9%)	37 (50.0%)	
BMI category			0.824
<25 kg/m^2^	15 (21.4%)	17 (23.0%)	
≥25 kg/m^2^	55 (78.6%)	57 (77.0%)	
pre-NAST axillary morphology (US-based)			**0.010**
<4 and non-conglomerate	25 (35.7%)	43 (58.1%)	
≥4 and non-conglomerate	28 (40.0%)	24 (32.4%)	
Conglomerate/matted nodes	17 (24.3%)	7 (9.5%)	
Tumor localization			0.777
Central–retroareolar	11 (15.7%)	12 (16.2%)	
Inner quadrant	10 (14.3%)	15 (20.3%)	
Outer quadrant	37 (52.9%)	34 (45.9%)	
Multicentric/diffuse	12 (17.1%)	13 (17.6%)	
Tumor side			0.338
Right	32 (45.7%)	28 (37.8%)	
Left	38 (54.3%)	46 (62.2%)	
Clinical T-stage and tumor extension			0.210
cT1-2	53 (75.7%)	49 (66.2%)	
cT3-4/extensive	17 (24.3%)	25 (33.8%)	
Histological grade			0.185
Grade 1–2	38 (54.3%)	32 (43.2%)	
Grade 3	32 (45.7%)	42 (56.8%)	
Tumor type			0.112 *
NST or other types	67 (95.7%)	74 (100%)	
Favorable histology	3 (4.3%)	0 (0.0%)	
Ki67 category			0.871
Low or intermediate	35 (50.0%)	36 (48.6%)	
High (≥30%)	35 (50.0%)	38 (51.4%)	
ER status			**0.001**
Negative or low-positive	9 (12.9%)	27 (36.5%)	
High-positive (≥10%)	61 (87.1%)	47 (63.5%)	
PR status			**0.002**
Negative or low-positive	22 (31.4%)	42 (56.8%)	
High-positive (≥20%)	48 (68.6%)	32 (43.2%)	
HR status			**<0.001**
Negative or low-positive	8 (11.4%)	27 (36.5%)	
High-positive	62 (88.6%)	47 (63.5%)	
HER2 status			**<0.001**
Negative (or low positive)	59 (84.3%)	40 (54.1%)	
Positive	11 (15.7%)	34 (45.9%)	
TNBC status			**0.007**
Non-TNBC	67 (95.7%)	60 (81.1%)	
TNBC	3 (4.3%)	14 (18.9%)	
Surrogate intrinsic subtype			**<0.001**
Luminal A	12 (17.1%)	7 (9.5%)	
Luminal B (HER2−)	44 (62.9%)	19 (25.7%)	
Luminal B (HER2+)	9 (12.9%)	24 (32.4%)	
HER2-enriched	2 (2.9%)	10 (13.5%)	
TNBC	3 (4.3%)	14 (18.9%)	
Residual tumor (ypT)			**<0.001**
ypT0	7 (10.0%)	38 (51.4%)	
ypT1-2	54 (77.1%)	32 (43.2%)	
ypT3-4	9 (12.9%)	4 (5.4%)	
In situ tumor (yp)			<0.001 *
No residual tumor	7 (10.0%)	35 (47.3%)	
Only residual in situ tumor	0 (0.0%)	3 (4.1%)	
Only residual invasive tumor	25 (35.7%)	20 (27.0%)	
Both in situ and invasive tumor	38 (54.3%)	17 (21.6%)	
Multifocality, multicentricity or satellite (yp)			0.110 *
None (already)	52 (74.3%)	58 (78.4%)	
Resolved or decreased	15 (21.4%)	16 (21.6%)	
Persisting	3 (4.3%)	0 (0.0%)	
Estimated tumor size reduction			**<0.001**
Complete response (ypT0)	7 (10.0%)	38 (51.4%)	
30–99% reduction	37 (52.9%)	29 (39.2%)	
<30% reduction or stable	15 (21.4%)	5 (6.8%)	
Progression	11 (15.7%)	2 (2.7%)	
PET/CT response (general; breast and axilla)			**<0.001**
Complete response	17 (24.3%)	41 (55.4%)	
Partial, stable, progression	53 (75.7%)	33 (44.6%)	
Modified PERCIST reduction			<0.001 *
Complete Response	20 (28.6%)	45 (60.8%)	
Partial Metabolic Response	43 (61.4%)	26 (35.1%)	
Stable Metabolic Disease	7 (10.0%)	2 (2.7%)	
Progressive Disease	0 (0.0%)	1 (1.4%)	
Breast Surgery			0.782
Breast-conserving surgery	12 (17.1%)	14 (18.9%)	
Mastectomy	58 (82.9%)	60 (81.1%)	

Note: *p*-values are derived from two-tailed tests. Fisher’s exact test was applied when expected cell counts were low. * Fisher’s exact test. Statistically significant results (*p* < 0.05) are indicated in bold. Surrogate intrinsic subtype assigned by ER/PR/HER2 and Ki-67 according to standard IHC surrogates. Abbreviations: BMI, body mass index; NST, invasive carcinoma of no special type; ER, estrogen receptor; PR, progesterone receptor; HR, hormone receptor; HER2, human epidermal growth factor receptor 2; US, ultrasound; TNBC, triple-negative breast cancer; yp, post-NAST; NAST, neoadjuvant systemic therapy; PET/CT, fluorodeoxyglucose positron emission tomography–computed tomography; estimated tumor size reduction (%) = [(Baseline tumor size − Residual tumor size post-NAST)/Baseline tumor size] × 100; Modified PERCIST (SUVmax-based) reduction (%), percent reduction in primary tumor SUVmax between pre-NAST and post-NAST PET/CT.

**Table 2:** Comparison of continuous clinicopathologic and PET/CT parameters according to axillary response status (non-ypN0 vs. ypN0).

Variables	Non-ypN0 (*n* = 70)	ypN0 (*n* = 74)	*p*-Value
Age (years)	52.53 (12.44)	51.45 (12.40)	0.602
pre-NAST axillary LN (mm)	20.0 (15.0–28.3)	16.0 (12.8–36.8)	**0.004**
pre-NAST breast tumor (mm)	35.0 (24.9–47.3)	30.0 (23.8–40.0)	0.423
ER (%)	90.0 (67.5–100.0)	50.0 (0.0–90.0)	**<0.001**
PR (%)	40.0 (8.8–80.0)	5.0 (0.0–52.5)	**0.001**
Ki67 (%)	27.5 (15.0–50.0)	30.0 (15.0–50.0)	0.724
HRSum (%)	130.0 (88.8–180.0)	85.5 (4.3–130.0)	**<0.001**
Residual Tumor (ypT, mm)	19.0 (6.0–31.0)	0.0 (0.0–15.0)	**<0.001**
Estimated Tumor Size Reduction (%)	40.8 (11.7–77.4)	100.0 (50.2–100.0)	**<0.001**
pre-NAST PET/CT Tumor	10.6 (7.4–16.2)	14.4 (9.6–21.9)	**0.003**
pre-NAST PET/CT Axilla	8.8 (5.5–13.8)	6.4 (2.2–12.5)	**0.014**
pre-NAST PET/CT Tumor-to-Axilla Ratio	1.2 (0.8–1.7)	2.0 (1.1–6.6)	**<0.001**
post-NAST PET/CT Tumor	3.3 (1.8–4.3)	1.3 (1.0–3.7)	**0.004**
post-NAST PET/CT Axilla	1.2 (1.0–3.0)	1.1 (1.0–1.1)	**0.001**
Modified PERCIST reduction	73.3 (55.6–83.5)	86.9 (68.5–92.6)	**<0.001**
SUVmax Response Delta (axillary)	0.8 (0.6–0.9)	0.8 (0.5–0.9)	0.821
Modified PET_MB Ratio	0.2 (0.1–0.5)	0.2 (0.1–0.3)	**0.002**
CA 15-3 (U/mL)	16.9 (12.7–23.4)	14.5 (9.7–19.3)	**0.024**
Delta IG (%)	−0.1 (−0.2–0.1)	0.0 (−0.2–0.2)	0.223
Albumin ratio	0.9 (0.9–1.0)	0.9 (0.9–1.0)	0.895
NLR	2.2 (1.7–2.6)	2.0 (1.7–2.7)	0.479
MLR	0.2 (0.2–0.3)	0.2 (0.2–0.3)	0.529
PLR	137.1 (104.6–163.7)	137.8 (103.3–166.7)	0.774
SII	564.9 (441.1–776.4)	545.9 (431.1–717.0)	0.949
PNI	53.3 (51.0–56.5)	54.7 (51.8–57.8)	0.142
PIV	273.9 (176.7–389.7)	257.1 (194.6–409.1)	0.804
HALP	39.3 (33.4–55.2)	41.9 (33.3–56.8)	0.589
HRR	0.92 (0.16)	0.95 (0.16)	0.184
MPVPLT	0.04 (0.03–0.05)	0.04 (0.03–0.04)	0.074

Note: Data are presented as median (interquartile range, IQR) for non-normally distributed variables and mean ± standard deviation (SD) for normally distributed variables. Statistically significant results (*p* < 0.05) are indicated in bold. *p*-values are derived from two-tailed tests. “Residual Tumor (ypT, mm)” denotes the size (mm) of the largest invasive focus in the breast on final pathology after NAST. “pre-NAST PET/CT Tumor” and “post-NAST PET/CT Tumor” denote the primary breast tumor SUVmax on pre- and post-NAST PET/CT, respectively, whereas “pre-NAST PET/CT Axilla” and “post-NAST PET/CT Axilla” denote the SUVmax of the index axillary lymph node (the node with highest FDG uptake). Abbreviations: LN, lymph node; ER, estrogen receptor; PR, progesterone receptor; HR, hormone receptor; HRSum (%), ER% + PR%; yp, post-NAST; NAST, neoadjuvant systemic therapy; PET/CT, fluorodeoxyglucose positron emission tomography–computed tomography; SUVmax, maximum standardized uptake value; CA 15-3, cancer antigen 15-3; IG, immature granulocyte; NLR, neutrophil-to-lymphocyte ratio; MLR, monocyte-to-lymphocyte ratio; PLR, platelet-to-lymphocyte ratio; SII, systemic immune-inflammation index; PNI, prognostic nutritional index; PIV, pan-immune-inflammation value; HALP, hemoglobin, albumin, lymphocyte, platelet score; HRR, hemoglobin-to-RDW ratio; MPVPLT, mean platelet volume-to-platelet ratio; estimated tumor size reduction (%) = [(Baseline tumor size − Residual tumor size post-NAST)/Baseline tumor size] × 100; tumor-to-axilla ratio: tumor SUVmax/axilla SUVmax; Modified PERCIST (SUVmax-based) reduction (%), percent reduction in primary tumor SUVmax between pre-NAST and post-NAST PET/CT; SUVmax Response Delta (axillary) = (pre-NAST Axilla SUVmax − post-NAST Axilla SUVmax)/pre-NAST Axilla SUVmax; Modified PET_MB Ratio = (post-NAST [tumor SUVmax + axilla SUVmax])/(pre-NAST [tumor SUVmax + axilla SUVmax]).

**Table 3:** ROC curve analysis for prediction of negative axillary status (ypN0) with optimal cut-off values.

Variables	AUC	95% CI	Optimal Cut-Off	Sensitivity (%)	Specificity (%)	*p*-Value
pre-NAST LN size (mm)	0.639	0.549–0.730	<19.35	63.5	57.1	**0.004**
ER (%)	0.704	0.619–0.789	<92.50	81.1	42.9	**<0.001**
PR (%)	0.656	0.567–0.746	<45.00	68.9	51.4	**0.001**
HRSum (%)	0.685	0.597–0.772	<100.50	64.9	34.3	**<0.001**
Residual tumor (ypT, mm)	0.752	0.672–0.833	<0.50	51.4	90.0	**<0.001**
Estimated Tumor Size Reduction (%)	0.771	0.693–0.848	≥40.83	86.5	50.0	**<0.001**
pre-NAST PET/CT Tumor	0.641	0.551–0.732	≥10.17	73.0	48.6	**0.003**
pre-NAST PET/CT Axilla	0.619	0.526–0.711	<8.76	64.9	48.6	**0.014**
pre-NAST PET/CT Tumor-to-Axilla Ratio	0.705	0.620–0.789	≥1.21	70.3	50.0	**<0.001**
post-NAST PET/CT Tumor	0.637	0.544–0.730	<2.65	63.5	67.1	**0.005**
post-NAST PET/CT Axilla	0.659	0.569–0.750	<1.85	89.2	42.9	**0.001**
Modified PERCIST reduction	0.694	0.607–0.781	≥80.70	64.9	67.1	**<0.001**
Modified PET_MB Ratio	0.647	0.557–0.737	<0.33	78.0	60.0	**0.002**
CA 15-3 (U/mL)	0.609	0.517–0.701	<15.15	55.4	60.0	**0.024**

Note: Statistically significant *p*-values (<0.05) are indicated in bold. *p*-values are derived from two-tailed tests. PET/CT variables use the same definitions as in Table 2: “Tumor” refers to primary breast tumor SUVmax, “Axilla” to index axillary lymph node SUVmax, and “Tumor-to-Axilla Ratio” to primary tumor SUVmax divided by index-node SUVmax. Abbreviations: ROC, receiver operating characteristic; AUC, area under the curve; CI, confidence interval; ER, estrogen receptor; PR, progesterone receptor; HR, hormone receptor; HRSum (%), ER% + PR%; yp, post-neoadjuvant; NAST, neoadjuvant systemic therapy; CA 15-3, cancer antigen 15-3; estimated tumor size reduction (%) = [(Baseline tumor size − Residual tumor size post-NAST)/Baseline tumor size] × 100; PET/CT, fluorodeoxyglucose positron emission tomography–computed tomography; SUVmax, maximum standardized uptake value measured by PET/CT; tumor-to-axilla ratio: tumor SUVmax/axilla SUVmax; Modified PERCIST (SUVmax-based) reduction (%), percent reduction in primary tumor SUVmax between pre-NAST and post-NAST PET/CT; Modified PET_MB Ratio = (post-NAST [tumor SUVmax + axilla SUVmax])/(pre-NAST [tumor SUVmax + axilla SUVmax]).

**Table 4:** Categorical analysis of ROC-derived predictors of axillary response status following neoadjuvant therapy (non-ypN0 vs. ypN0).

Variables	Non-ypN0 (*n* = 70)	ypN0 (*n* = 74)	*p*-Value
pre-NAST axillary LN Size (mm)			**0.013**
<19.35	30 (42.9%)	47 (63.5%)	
≥19.35	40 (57.1%)	27 (36.5%)	
ER (%)			**0.002**
<92.50	40 (57.1%)	60 (81.1%)	
≥92.50	30 (42.9%)	14 (18.9%)	
PR (%)			**0.032**
<45	36 (51.4%)	51 (68.9%)	
≥45	34 (48.6%)	23 (31.1%)	
HRSum (%)			**<0.001**
Low (≤100.5)	24 (34.3%)	48 (64.9%)	
High (>100.5)	46 (65.7%)	26 (35.1%)	
pre-NAST PET/CT Tumor			**0.008**
<10.17	34 (48.6%)	20 (27.0%)	
≥10.17	36 (51.4%)	54 (73.0%)	
pre-NAST PET/CT Axilla			**0.048**
<8.76	34 (48.6%)	48 (64.9%)	
≥8.76	36 (51.4%)	26 (35.1%)	
pre-NAST PET/CT Tumor-to-Axilla Ratio			**0.013**
<1.21	35 (50.0%)	22 (29.7%)	
≥1.21	35 (50.0%)	52 (70.3%)	
post-NAST PET/CT Tumor			**<0.001**
<2.65	23 (32.9%)	27 (36.5%)	
≥2.65	47 (67.1%)	47 (63.5%)	
post-NAST PET/CT Axilla			**<0.001**
<1.85	40 (57.1%)	65 (87.8%)	
≥1.85	30 (42.9%)	9 (12.2%)	
Residual Tumor Size (ypT, mm)			**<0.001**
<0.5 mm	7 (10.0%)	38 (51.4%)	
≥0.5 mm	63 (90.0%)	36 (48.6%)	
Estimated Tumor Size Reduction (%)			**<0.001**
<40.83	35 (50.0%)	10 (13.5%)	
≥40.83	35 (50.0%)	64 (86.5%)	
Modified PERCIST reduction			**<0.001**
Low Reduction (<80.7%)	47 (67.1%)	26 (35.1%)	
High Reduction (≥80.7%)	23 (32.9%)	50 (64.9%)	
Modified PET_MB Ratio			**0.017**
Low (<0.33)	42 (60.0%)	58 (78.4%)	
High (≥0.33)	28 (40.0%)	16 (21.6%)	
CA 15-3 (U/mL)			0.064
Low (<15.15)	28 (40.0%)	41 (55.4%)	
High (≥15.15)	42 (60.0%)	33 (44.6%)	

Note: Statistically significant results (*p* < 0.05) are indicated in bold. *p*-values are derived from two-tailed tests. PET/CT variables use the same definitions as in Table 2: “Tumor” refers to primary breast tumor SUVmax, “Axilla” to index axillary lymph node SUVmax, and “Tumor-to-Axilla Ratio” to primary tumor SUVmax divided by index-node SUVmax. Abbreviations: ROC, receiver operating characteristic; ER, estrogen receptor; PR, progesterone receptor; HR, hormone receptor; HRSum (%), ER% + PR%; CA 15-3, cancer antigen 15-3; yp, post-NAST; NAST, neoadjuvant systemic therapy; PET/CT, fluorodeoxyglucose positron emission tomography–computed tomography; SUVmax, maximum standardized uptake value; tumor-to-axilla ratio: tumor SUVmax/axilla SUVmax; estimated tumor size reduction (%) = [(Baseline tumor size − Residual tumor size post-NAST)/Baseline tumor size] × 100; Modified PERCIST (SUVmax-based) reduction (%), percent reduction in primary tumor SUVmax between pre-NAST and post-NAST PET/CT; Modified PET_MB Ratio: (post-NAST [tumor SUVmax + axilla SUVmax])/(pre-NAST [tumor SUVmax + axilla SUVmax]).

**Table 5:** Multivariable logistic regression model predicting ypN0 axillary response.

Predictor	Odds Ratio (OR)	95% CI	*p*-Value
pre-NAST axillary LN morphology (US-based)			
≥4 non-conglomerate vs. <4 non-conglomerate	0.56	0.22–1.41	0.216
conglomerate/matted vs. <4 non-conglomerate	0.13	0.04–0.51	**0.003**
Residual Tumor Size, ypT < 0.5 mm (vs. ≥0.5 mm)	10.63	3.51–32.17	**<0.001**
TNBC (yes vs. no)	11.41	2.43–53.54	**0.002**
pre-NAST PET/CT Tumor-to-Axilla Ratio (≥1.21)	2.76	1.13–6.74	**0.026**
Modified PERCIST reduction (≥80.70%)	3.56	1.46–8.69	**0.005**

Note: Statistically significant results (*p* < 0.05) are indicated in bold. *p*-values are derived from two-tailed tests. Two-sided *p* values are reported. Omnibus model χ^2^ = 64.74 (df = 6), *p* < 0.001; Hosmer–Lemeshow goodness-of-fit test χ^2^ = 8.09 (df = 8), *p* = 0.425; Nagelkerke R^2^ = 0.483; overall correct classification rate = 76.4%. model AUC = 0.857 (95% CI 0.797–0.917; SE 0.031; *p* < 0.001). Pre-NAST axillary morphology (US-based) was modeled as a three-level factor (<4 non-conglomerate [reference], ≥4 non-conglomerate, conglomerate/matted). Abbreviations: CI, Confidence Interval; LN, lymph node; yp, post-NAST; NAST, neoadjuvant systemic therapy; US, ultrasound; TNBC, triple-negative breast cancer; PET/CT, fluorodeoxyglucose positron emission tomography–computed tomography; SUVmax, maximum standardized uptake value measured by FDG PET/CT; Modified PERCIST (SUVmax-based) reduction (%), percent reduction in primary tumor SUVmax between pre-NAST and post-NAST PET/CT.

**Table 6:** Nomogram points for predicting axillary pathological complete response (ypN0).

Variable	Category	Points
TNBC	yes	+100
Residual Tumor Size (ypT)	<0.5 mm	+97
Modified PERCIST reduction	high (≥80.70%)	+52
pre-NAST PET/CT Tumor-to-Axilla Ratio	≥1.21	+42
pre-NAST axillary morphology (US-based)	conglomerate/matted	−83
	4 or more nodes and non-conglomerate	−24

Note: Interpretation of the total nomogram score used for predicting the probability of axillary pathological complete response (ypN0): ≤−28 (<10%), −27 to 28 (10–30%), 29 to 62 (31–50%), 63 to 97 (51–70%), 98 to 134 (71–85%), and >134 (>85%). Higher total scores indicate greater probability of ypN0; negative totals reflect reduced probability, and positive totals reflect increased probability. Pre-NAST axillary morphology categories correspond to the three-level factor used in Table 5 (<4 non-conglomerate, ≥4 non-conglomerate, conglomerate/matted). Abbreviations: yp, post-NAST; NAST, neoadjuvant systemic therapy; TNBC, triple-negative breast cancer; PET/CT, fluorodeoxyglucose positron emission tomography–computed tomography; SUVmax, maximum standardized uptake value measured by FDG PET/CT; Modified PERCIST (SUVmax-based) reduction (%), percent reduction in primary tumor SUVmax between pre-NAST and post-NAST PET/CT; US, ultrasound.

## Data Availability

De-identified data are available from the corresponding author upon reasonable request and institutional approval.

## Abbreviations

ALN, axillary lymph node; ALND, axillary lymph node dissection; AUC, area under the curve; BMI, body mass index; CA 15-3, cancer antigen 15-3; CI, confidence interval; DCIS, ductal carcinoma in situ; ECE, extracapsular extension; HRR, hemoglobin-to-RDW ratio; IG, immature granulocyte; HALP, hemoglobin, albumin, lymphocyte, platelet score; ER, estrogen receptor; estimated tumor size reduction (%) = [(Baseline tumor size − Residual tumor size post-NAST)/Baseline tumor size] × 100; HR, hormone receptor (ER% + PR%); HER2, human epidermal growth factor receptor 2; LN, lymph node; MLR, monocyte-to-lymphocyte ratio; MMG, mammography; MRI, magnetic resonance imaging; Modified PET_MB Ratio, Modified Total Metabolic Burden Ratio = (post-NAST [tumor SUVmax + axilla SUVmax])/(pre-NAST [tumor SUVmax + axilla SUVmax]); MPVPLT, mean platelet volume-to-platelet ratio; NAST, neoadjuvant systemic therapy; NLR, neutrophil-to-lymphocyte ratio; non-cong., non-conglomerate; SII, systemic immune-inflammation index; NST, invasive carcinoma of no special type; pCR, pathological complete response; PET/CT, fluorodeoxyglucose positron emission tomography–computed tomography; Modified PERCIST (SUVmax-based) reduction (%), percent reduction in primary tumor SUVmax between pre-NAST and post-NAST PET/CT; ROC, receiver operating characteristic; SUVmax Response Delta (axillary) = (pre-NAST Axilla SUVmax − post-NAST Axilla SUVmax)/pre-NAST Axilla SUVmax; PIV, pan-immune-inflammation value; PLR, platelet-to-lymphocyte ratio; PNI, prognostic nutritional index; PR, progesterone receptor; RT, radiotherapy; SLN, sentinel lymph node; SLNB, sentinel lymph node biopsy; SUVmax, maximum standardized uptake value; TNBC, triple-negative breast cancer; US, ultrasound; yp, post-NAST.
